# The importance of using whole genome sequencing and extended spectrum beta-lactamase selective media when monitoring antimicrobial resistance

**DOI:** 10.1038/s41598-020-76877-7

**Published:** 2020-11-16

**Authors:** Nicholas Duggett, Manal AbuOun, Luke Randall, Robert Horton, Fabrizio Lemma, Jon Rogers, Derrick Crook, Christopher Teale, Muna F. Anjum

**Affiliations:** 1 Animal and Plant Health Agency, Weybridge, New Haw, Addlestone, KT15 3NB Surrey UK; 2 Nuffield Department of Medicine, University of Oxford, Oxford, UK; 3 NIHR Health Protection Research Unit in Healthcare Associated Infections and Antimicrobial Resistance at University of Oxford in Partnership With Public Health England, Oxford, UK; 4 NIHR Oxford Biomedical Research Centre, JR Hospital, Oxford, UK; 5 Present Address: School of Health and Life Sciences, Teesside University, Middlesbrough, TS1 3BX UK

**Keywords:** Computational biology and bioinformatics, Microbiology, Molecular biology, Diseases, Pathogenesis

## Abstract

To tackle the problem of antimicrobial resistance (AMR) surveillance programmes are in place within Europe applying phenotypic methods, but there are plans for implementing whole genome sequencing (WGS). We tested the benefits of WGS using *Escherichia coli* collected from pig surveillance performed between 2013 to 2017. WGS was performed on 498 *E. coli* producing ESBL and AmpC enzymes, recovered from pig caeca on MacConkey + cefotaxime (McC + CTX) agar, as recommended by the European Commission, or ESBL agar, used additionally by United Kingdom. Our results indicated WGS was extremely useful for monitoring trends for specific ESBL genes, as well as a plethora of AMR genotypes, helping to establish their prevalence and co-linkage to certain plasmids. Recovery of isolates with multi-drug resistance (MDR) genotypes was lower from McC + CTX than ESBL agar. The most widespread ESBL genes belonged to the *bla*_CTX-M_ family. *bla*_CTX-M-1_ dominated all years, and was common in two highly stable IncI1 MDR plasmids harbouring (*bla*_CTX-M-1_,*sul2*, *tetA*) or (*bla*_CTX-M-1_, *aadA5*, *sul2*, *dfrA17*), in isolates which were phylogenetically dissimilar, suggesting plasmid transmission. Therefore, WGS provided a wealth of data on prevalence of AMR genotypes and plasmid persistence absent from phenotypic data and, also, demonstrated the importance of culture media for detecting ESBL *E. coli*.

## Introduction

Increasing levels of antimicrobial resistance (AMR) is a global concern to both human and animal health. The O’Neill report estimated 10 million lives will be at risk per year due to resistant infections by 2050 if policies to stop the spread of AMR are not implemented^1^. The One Health concept requires a holistic approach to looking at AMR in humans, animals and the environment. Therefore, gaining information on AMR isolates in indicator species, such as commensal *E. coli* from livestock, is invaluable to characterise the resistome and track any emerging trends through the food chain. As WGS has become a more widely utilised resource, EU Agencies such as EFSA and ECDC have proposed that it is used by EU member states in surveillance activities whether it be harmonised AMR surveillance^2^, or for outbreak investigations^3^.

The WHO has identified and ranked antimicrobials by those deemed critically important for human medicine^4^. This list includes third and higher generation cephalosporins, which are targeted by bacterial production of extended spectrum beta-lactamase (ESBL) and AmpC enzymes, and *E. coli* harbouring these genes are the subject of specific surveillance by EU members, which is monitored by EFSA. These antibiotics have been used in human medicine since the early 1980s with resistance to extended-spectrum cephalosporins (ESCs) first noted in *Klebsiella pneumoniae* and *Serratia marcesens* in 1983, rising rapidly in the intervening years^5,6^. One of the primary drivers behind this increase in prevalence of resistant isolates is the occurrence of promiscuous plasmids harbouring *bla*_CTX-M_ genes, which are often co-located with other resistance genes, found in Enterobacteriaceae^5^. Plasmid types harbouring ESBL genes vary, but frequently include plasmids of incompatibility groups I1, and F (IncI1 and IncF). ESBL-producing *E.coli* have been isolated from multiple sources including healthy humans, livestock and companion animals, in addition to food-stuffs, and are the most common bacterial hosts of acquired *bla*_CTX-M_^7–13^_._ ESBL harbouring *E. coli* can belong to different STs with ST131 most commonly associated with infection in humans and which has rarely been identified in livestock; others such as ST10 and ST88 have been found in both humans and animals^7,8,11,14^.

The aim of this study was to perform molecular characterisation using WGS to determine the benefits of using this approach for surveillance. We expected to identify AMR genes and circulating plasmids in *E. coli* isolated on selective agars containing ESCs. These were collected from porcine caecal samples from healthy pigs from a UK nationwide pig study in 2013, and EU mandatory monitoring of healthy pigs at slaughter in the UK in 2015 and 2017; each caecal content was cultured on two different ESC containing agar plates. Although the isolates from 2013 had previously been analysed using phenotypic methods^15^, they had not been genotyped and so are included here for comparison with datasets from 2015 and 2017. A secondary aim of the study was to compare isolates from the EU monitoring agar, MacConkey + 1 mg/L cefotaxime with an additional ESBL agar, to determine any benefits from using an additional agar. AMR genotypes of isolates were characterised in detail through the APHA SeqFinder WGS pipeline that we have validated for use in AMR surveillance^16^. Selected plasmids carrying AMR genes, including MDR plasmids, were characterised by long read sequencing, and compared to those from NCBI and other studies to look at transmission and persistence.

## Results

### Antimicrobial resistance identified in *E. coli* isolates from EU and national surveillance

Genotypic profiles were established through WGS of *E. coli* producing ESBL or AmpC enzymes (Supplementary Table S1) isolated in 2015 and 2017 from EU surveillance on two agar plates, one specified by EFSA (McC + CTX) and an additional plate (ESBL) supplemented by UK for national surveillance activities in livestock, which are reported through VARSS^17,18^. Moreover, WGS of *E. coli* collected through national surveillance of pigs in 2013 selected on ESBL agars have also been included for investigation of trends over five years. From the McC + CTX isolates, overall there was a ~ 97% correlation between the phenotype (based on ECOFFs) and the resistance genotype, with the majority of antimicrobial classes showing excellent to good correlations (Supplementary Table S3). The discordance was primarily attributed to azithromycin over-reporting, because the presence of *mphA* does not always result in an azithromycin MIC above the cut-off^19^. In addition, the presence of a *tet(A)* variant that was initially not in our database resulted in non-concordance between the genotype and phenotype of five isolates. Once this gene was added there was a 100% correlation for tetracycline phenotypic and genotypic resistance, increasing the overall correlation to ~ 98%.

#### AMR genotypes in isolates recovered on McC + CTX agar from 2015 and 2017 EU surveillance

**Figure 1:**
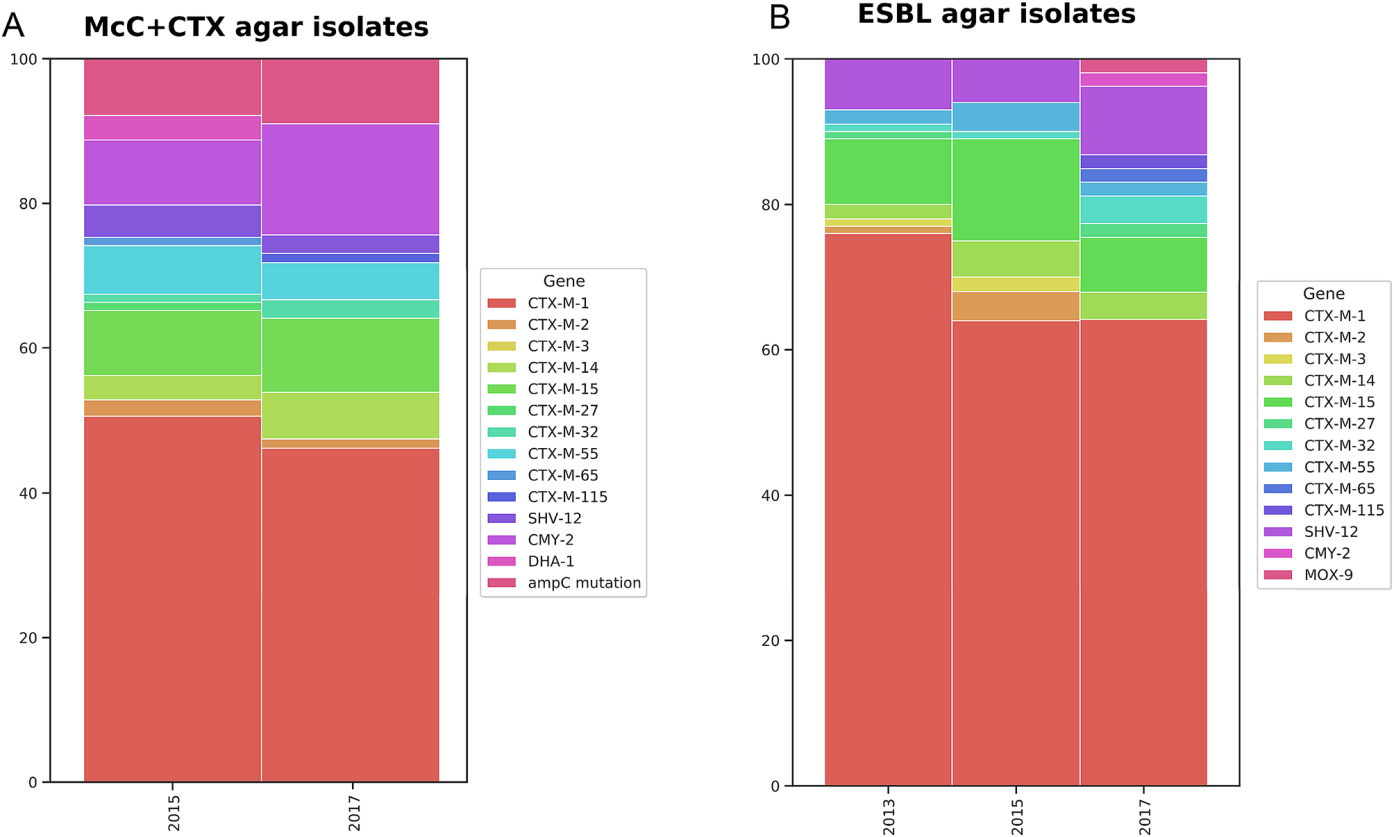
Differing proportions of AmpC/ESBL genes were present in isolates from McC + CTX (**A**) or ESBL (**B**) agar collected in 2013, 2015 and 2017.

Eighty-nine isolates were recovered from 313 caecal samples on McC + CTX agar in 2015, and 75 from 347 caecal samples in 2017; as expected all isolates harboured resistance genes to ESCs, and to other antimicrobials, including the ten others in the EFSA panel for *E. coli* outlined in Decision 2013/652/EU. A greater proportion of isolates in 2015 were identified as ESBLs compared to those in 2017, as more allelic variants of *bla*_CTX-M_ were present in 2015 (Fig. 1, Supplementary Table S2). *bla*_CMY-2_ and AmpC promoter mutations were found in isolates from both years from these plates and comprised 15/89 (17%) and 20/75 (27%) of the isolates recovered in 2015 and 2017 respectively; three isolates from 2015 contained *bla*_DHA-1,_ the only time point this gene was identified in this study. Genotypic resistance of isolates to the non-beta lactam antimicrobials present in the EFSA panel (Supplementary Table S3), as a proportion of the total isolates examined, was lower in 2017, except for trimethoprim; but this reduction was not statistically significant. Genotypic resistance to the highest non-beta-lactam antimicrobials, sulfamethoxazole, tetracycline and trimethoprim, in both years was most often attributed to the presence of *sul2*, *tet(A)* and *dfrA17*. Genes conferring resistance to five antimicrobials not in the EFSA panel were present in both years, with spectinomycin/streptomycin most frequently detected, primarily due to presence of *aadA* variants, *ant3-1a*, *strA* and *strB* (Table S3). Genotypic resistance to the critically important antimicrobial fosfomycin, conferred by *fosA3*, was identified in one isolate from 2015, and two isolates from 2017. The most common resistance gene combination in 2015 from the McC + CTX agar was *bla*_CTX-M-1_, *sul2* and *tet(A)* (4%), but in 2017 isolates AmpC promoter mutations were the most common (4%; Table 1).

**Table 1:** Most common genes conferring genotypic resistance per set of isolates and the proportion of isolates with that resistance gene pattern.

Year and agar type	Most common resistance gene allelic combination and proportion
2013 ESBL	*aadA5*, *bla*_CTX-M-1_, *sul2*, *dfrA17* (8%)
2015 ESBL	*bla*_CTX-M-1_, *sul2*, *tet(A)* (7%)
2015 McC + CTX	*bla*_CTX-M-1_, *sul2*, *tet(A)* (4%)
2017 ESBL	*ant3-1a*, *bla*_CTX-M-1_, *sat2A*, *sul2*, *tet(A)* (8%)
2017 McC + CTX	AmpC promoter mutation (4%)

**Figure 2:**
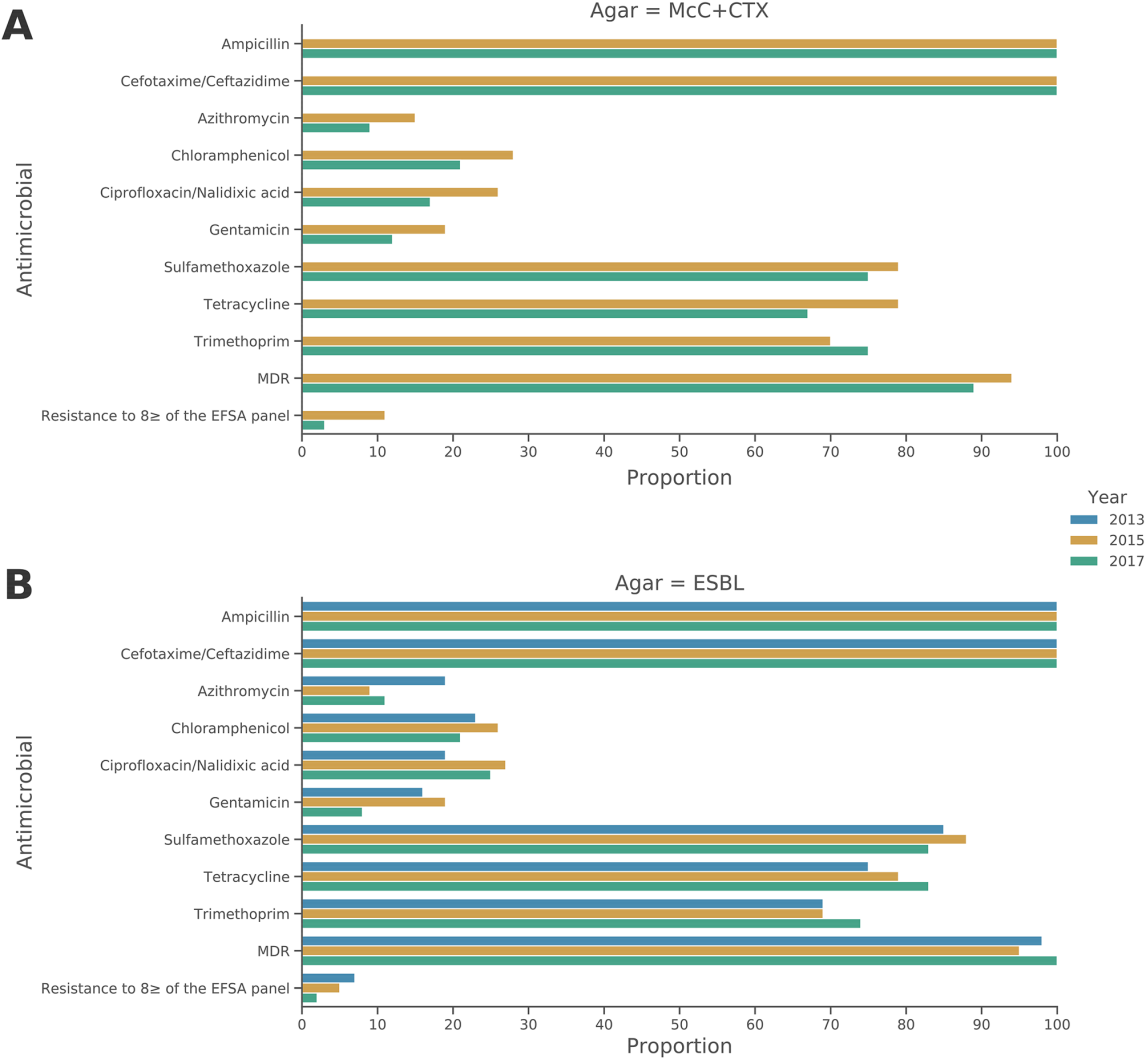
Proportion of resistance to antimicrobials present in the EFSA *E. coli* monitoring panel in isolates originating from the MacConkey + cefotaxime agar in 2015 and 2017 (**A**) and ESBL agars in 2013 (Chromagar CTX, ESBL Brilliance), 2015 (ESBL Brilliance) and 2017 (Chromagar ESBL; **B**).

**Figure 3:**
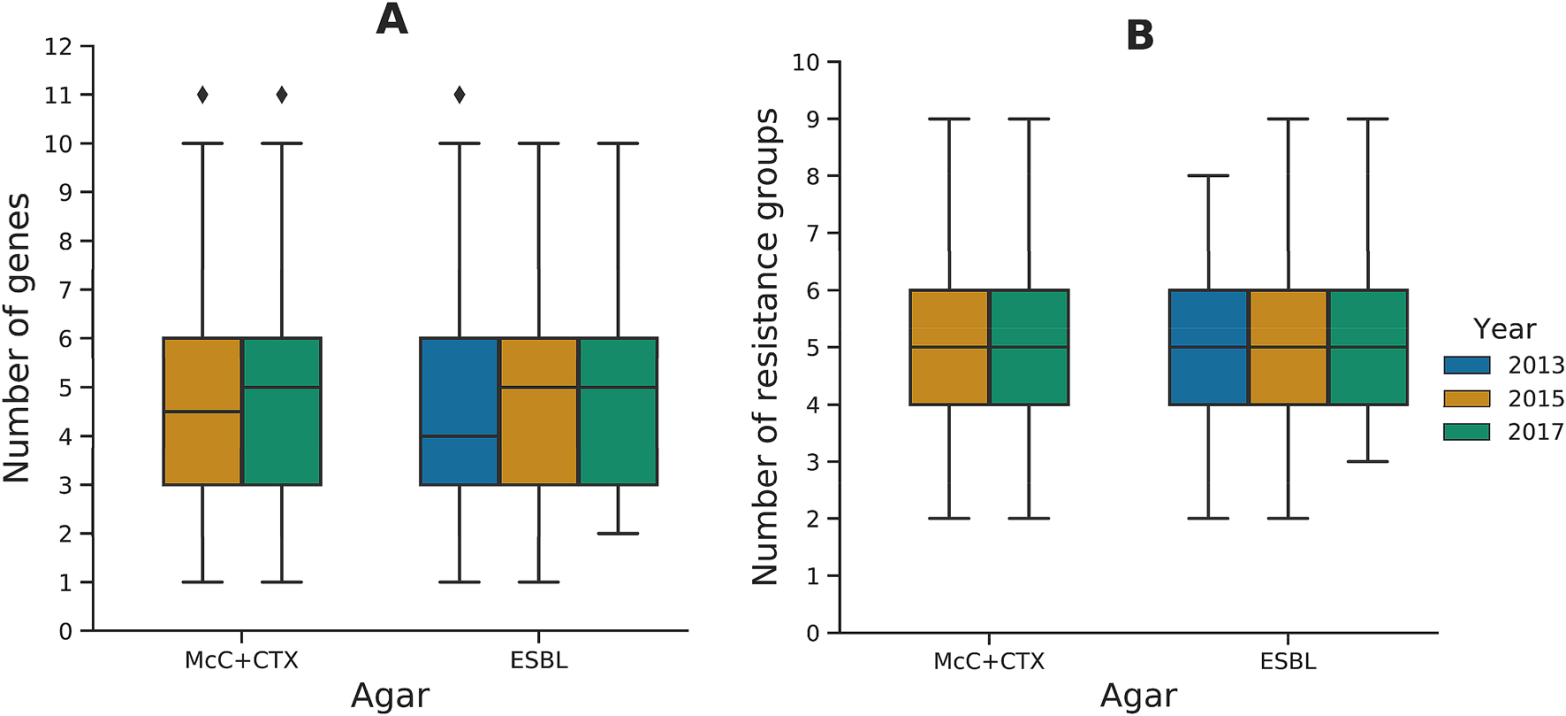
Boxplot of the number of acquired resistance genes per isolate conferring genotypic resistance to antimicrobials in the EFSA panel for the different agar types and years (**A**), and number of resistance groups in the EFSA panel per isolate (**B**).

The proportion of isolates with genotypic MDR i.e. presence of resistance genes to three or more AMR classes, was slightly higher in isolates from 2015 (94%), than for isolates from 2017 (89%; Fig. 2A, Supplementary Table S3) and these figures were the same as those calculated from the MIC phenotypic data available (not shown). Significantly (P =  < 0.05), the percentage of isolates that were resistant genotypically to eight or more of the EFSA panel were substantially reduced from 11 to 3%, in this period. The median number of resistance genes per isolate was five in 2017, compared to 4.5 in 2015 (Fig. 3), but the number of EFSA antimicrobial classes these genes conferred resistance to remained at five. Under EFSA MDR rules, ampicillin and cefotaxime/ceftazidime are treated as independent classes. However, because ESC genes confer resistance to both of these, an isolate harbouring a single ESC gene will count as having resistance to two classes therefore, sometimes elevating the median number of antimicrobial classes the isolate is resistant to beyond the number of resistance genes it harbours. *bla*_CTX-M-1_ was the most common ESC gene present in isolates from the 2015 and 2017 McC + CTX plate (Fig. 1A, Supplementary Table S2).

#### AMR genotypes in isolates recovered on ESBL agar from a 2013 national survey, and 2015, 2017 EU surveillance

More ESBL isolates were collected from the national survey (n = 185/637) in 2013, than in 2015 (n = 96/313) and 2017 (n = 53/347) from ESBL agar plates. Isolates were most likely to harbour *bla*_CTX-M-1_, which accounted for the highest proportion of ESBL genes detected in 2013 (76%, Fig. 1B, Supplementary Table S2). Overall, nine other *bla*_CTX-M_ variants were recovered from ESBL agar; 14% of isolates harboured *bla*_CTX-M-15_ in 2015; the only other non-*bla*_CTX-M_ variant ESBL gene identified was *bla*_SHV-12_ (Supplementary Table S2). Unexpectedly, *bla*_CMY-2_ and *bla*_MOX-9_ without an ESBL gene were present in one isolate each from 2017, indicating possible disruption of porins thus allowing the sample to grow on CHROMagar ESBL^20^. Mutations in the AmpC promoter region were identified in one isolate from each year; these isolates also harboured an ESBL gene. As with isolates recovered from McC + CTX agar, *sul2*, *tet(A)* and *dfrA17* were the most frequently detected non-beta-lactamases. Azithromycin resistance was highest in the 2013 ESBL isolates, and there were also a high proportion of isolates with resistance to the non-EFSA antimicrobial streptomycin/spectinomycin, conferred by *aadA* variants, *strA*, *strB* and *ant3-1a*, just as for the McC + CTX isolates. Genotypic fosfomycin resistance, attributed to *fosA3*, was identified in three isolates from 2013 and an isolate from 2015 ESBL agar. The most common gene combination in 2013 was *aadA5*, *bla*_CTX-M-1_, *sul2* and *dfrA17*, found in 8% of isolates. Both 2015 and 2017 isolates harboured *bla*_CTX-M-1_, *sul2* and *tet(A)*, but the most common genotype in 2017 also included *ant3-1a* and *sat2A* (Table 1). The median number of resistance genes in isolates from 2013 (n = 4) was slightly lower compared to 2015 and 2017 (n = 5); however the median number of resistances classes was five for all years. The reason for the number of genes being lower than the number of classes is due to ESBL and AmpC genes confer resistance to two EFSA classes: ampicillin and cefotaxime or ceftazidime. This is similar to the McC + CTX isolates where a level of gene redundancy was also observed (Fig. 3B).

The proportion of isolates demonstrating genotypic MDR in 2013 was 98%, which was higher than the 2015 ESBL isolates (94%) but lower than the 2017 ESBL isolates, where 100% of isolates were genotypically MDR. The 2013 and 2017 MDR proportions were higher than recovered from the McC + CTX agar, however, the 2015 ESBL and 2015 McC + CTX proportions were the same (Fig. 2B, Supplementary Table S3). Despite the fluctuation in genotypic MDR from 2013 to 2017, the ratio of isolates recovered from ESBL plates resistant to eight or more of the EFSA antimicrobial panel decreased from 7% in 2013 to 2% in 2017, however this was not a statistically significant reduction (Fig. 2B).

### Characterisation of plasmid genomes

Phenotypic characterisation provides no detail on plasmids most likely responsible for AMR transmission, which is an advantage of WGS. To aid their characterisation, long and short read WGS data were used to circularise eight plasmids from six isolates. Isolate selection was based upon either incomplete short-read plasmid data, or the ESBL/AmpC gene they possessed, so the most complete information on the plasmid host background could be obtained. Of the 498 ESBL/AmpC isolates included in this study with short-read WGS data available, 67% of isolates did not contain the same genes as those present on the circularised ESBL/ampC gene-harbouring plasmids with > 99% sequence similarity, indicating the immense genetic diversity of these plasmids.

The *bla*_CTX-M-1_ genes were primarily identified on IncI1-type ST3 plasmids, which formed two groups, similar to pPE13096 or pESBL138 (Table 1). pESBL138 *bla*_CTX-M-1_ IncI1-ST3 plasmids contained *sul2* and *tetA*, whereas pPE13096 *bla*_CTX-M-1_ was co-located with *aadA5*, *sul2* and *dfrA17* (Table 2; Supplementary Fig. S1a). The 108 kb pESBL138 was most similar to plasmids isolated from broilers in France in 2010–2012 (Supplementary Fig. S1b)^21^; and the 110 kb pPE13096, showed highest similarity to two plasmids of unknown French origin (GenBank accession: LT985235.1 and LT985286.1, Supplementary Fig. S1b). The two plasmid types were not related to a particular host *E. coli* ST. Using the resolved plasmid genomes as reference we noted 42 isolates to harbour > 99% average nucleotide identity to the genes of pPE13096, and 131 isolates to pESBL138, indicating likely presence of similar plasmids (Table 2).

**Table 2:** Plasmids that were circularised from hybrid short and long read sequencing with their Incompatibility type, associated AMR genes, size and number of isolates that shared > 99% similarity with the circularised plasmids.

Plasmid ID	Year host *E. coli* was isolated	Plasmid Inc-type	Associated AMR genes	Size (kb)	No. of isolates from all years with > 99% identity
pPE13096	2013	I1	*aadA5, bla*_CTX-M-1_*, sul2, dfrA17*	110	42
pPO125	2015	Y	*bla*_CTX-M-15_*, dfrA14, qnrS1, strA, strB, sul2, bla*_TEM-1b_*, tetA*	98	5
pPO189_X1	2015	X1	*dfrA14, lnuF, sul3, bla*_TEM-1b_*, tetA*	33	3
pPO189_F		F	*dfrA8, bla*_TEM-1b_*, strA, strB, sul2*	74	5
pESBL138	2015	I1	*bla*_CTX-M-1_*, sul2, tetA*	108	131
p3682_I1	2017	I1	*bla*_CMY-2_	91	1
p3682_FQ		F/Q	*ant3-Ia, catA1, dfrA1, mphB, bla*_TEM-1b_*, tetA, strA, strB, sul2, sul3*	169	1
pPO116	2017	X3	*bla*_SHV-12_*, qnrS1*	42	11

p3682_I1, a 91 kb IncI1 plasmid that harboured *bla*_CMY-2_ was only present in isolate 3682 (Table 2). The backbone of this plasmid was most similar to a plasmid isolated from a bovine *Salmonella* in USA which lacked *bla*_CMY-2_, and also from a Scottish canine *E. coli* with *bla*_CMY-2_ (Supplementary Fig. S2)^22^. A 169 kb MDR plasmid, p3682_FQ that contained both IncF and IncQ replicons was also identified in isolate 3682 (Table 2), indicating presence of a possible plasmid hybrid. Similar plasmids were not identified in NCBI, but it was comparable to other plasmids from ongoing APHA projects (data not shown), suggesting wider distribution.

The *bla*_CTX-M-15_ plasmid sequenced was pPO125 with an IncY replicon and multiple resistance genes (Table 2). This plasmid showed some similarity to two plasmids from the USA, including one that was isolated from a hospital plumbing system (Supplementary Fig. S3)^23^; and only showed sequence similarity with five other isolates in our collection. pP0116, a 42 kb IncX3 plasmid that contained *bla*_SHV-12_ and *qnrS1*, matched to 11 isolates from our study and showed high sequence similarity to other IncX3 plasmids from a human urinary-tract infection and chicken faeces isolated in the Netherlands in 2009 and 2014, respectively (Supplementary Fig. S4)^24^.

Hybrid assembly of isolate PO189 containing *bla*_CTX-M-55,_ *qnrS1* and *aac3-IVa* indicated that these genes had integrated into the same chromosomal region and the chromosomal location of *bla*_CTX-M-55_ and *qnrS1* was identified in ten isolates. Although isolate PO189 did not harbour an ESBL gene on a plasmid, it harboured two MDR plasmids (pPO189_X1 and pPO189_F), which were further identified in three and five isolates, respectively (Table 2). Both plasmids contained genes that conferred resistance to trimethoprim, sulphonamide and ampicillin, showing a level of redundancy in the host (Table 2).

### Diversity of *E. coli* genotypes

Another advantage of WGS is the ability to characterise the host genome harbouring AMR genes. In silico MLST performed on the isolates assigned 114 STs, in addition to 20 STs that were novel, indicating much diversity in the host. The most abundant STs were ST88 (n = 38), ST10 (n = 35) and ST101 (n = 35), but the dominant ST for each year fluctuated. Two isolates from 2017 were assigned to ST131, an *E. coli* ST associated with human infections. However, one isolate contained *bla*_CTX-M-1_ and the other contained *bla*_CTX-M-27_, rather than *bla*_CTX-M-15_ typically associated with the epidemic clone of ST131 in humans. Although the *bla*_CTX-M-1_ isolate harboured the *fimH22* allele, the *bla*_CTX-M-27_ isolate did harbour the *fimH30* allele, fluroquinolone resistance and the prophage region associated with the C1-M27 subclade associated with human pathogenic ST131 strains^25^.

**Figure 4:**
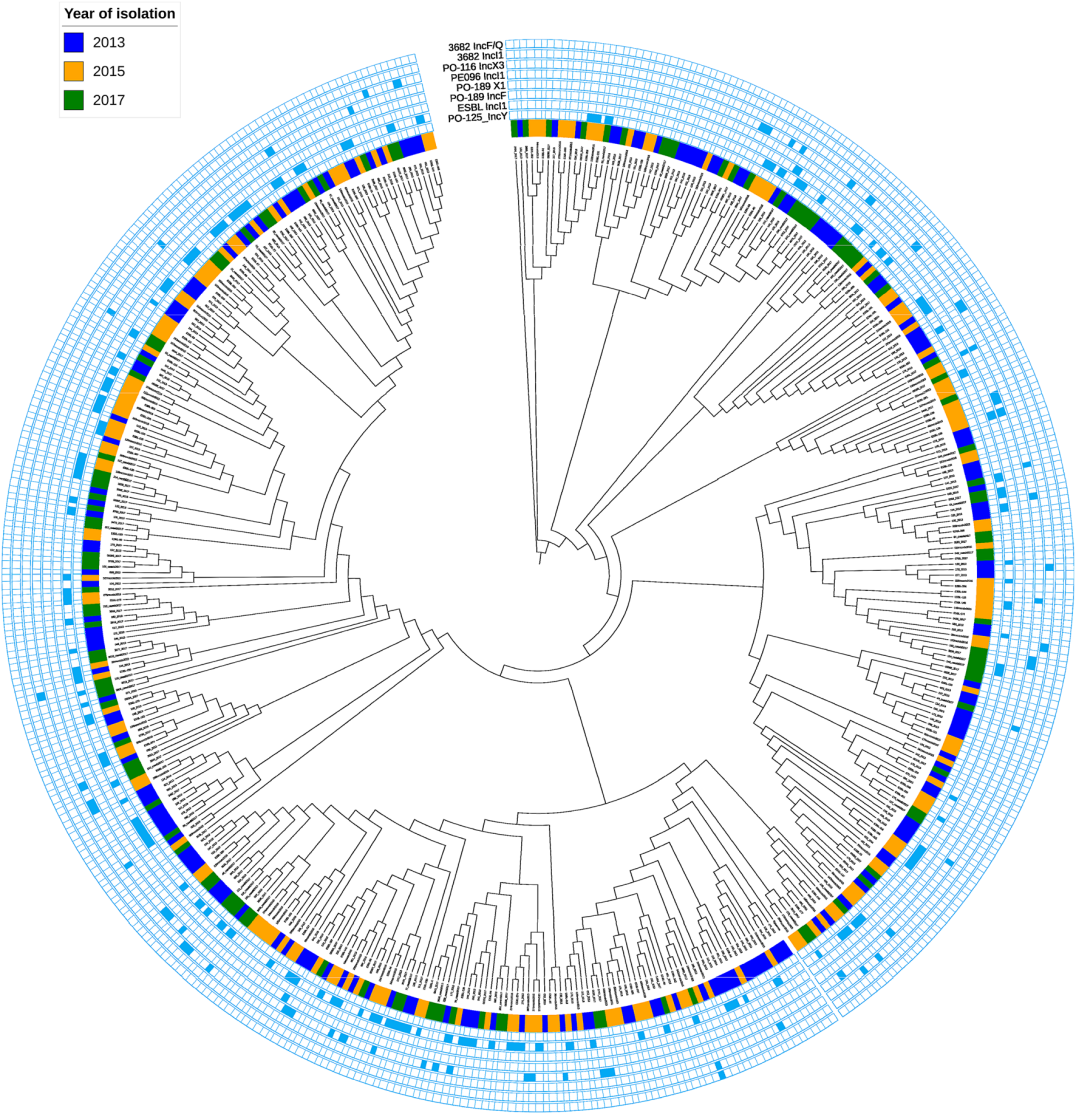
Phylogenetic tee of *E. coli* isolates from 2013, 2015 and 2017. The inner circle colours dictate the year the *E. coli* were isolated and the other rings show the presence of plasmids with high similarity to those listed in Table 2.

To establish any links between isolates from different years, a maximum-likelihood phylogenetic tree of the core-genome SNPs was constructed. The tree reiterated that the isolates recovered from each year were not clonal and did not cluster by year (Fig. 4). The isolates with > 99% match to the circularised plasmids were also mapped to the tree to determine their distribution. Isolates that contained either IncI1 *bla*_CTX-M-1_ plasmid types (pPE13096 or pESBL138), which was the most common plasmid type, were interspersed throughout the tree, indicating these plasmids were not restricted to a narrow *E. coli* host range.

## Discussion

The monitoring of isolates by WGS provides a wealth of data, as we have shown previously^16^, and in this paper there was very good correlation between resistance genotypes and resistance phenotypes determined by MICs and applying ECOFFs, which improved further on addition of a gene variant to our APHA SeqFinder AMR database. We believe this criterion applies widely, and demonstrates the need for continual assessment, and iterative improvement of WGS pipelines. Furthermore, as demonstrated here and previously^16^, for a small number of antimicrobial classes, gene presence was not always associated with a phenotype. Nevertheless, WGS is extremely useful for monitoring the trends of specific resistances and their underlying mechanism, in addition to tracking the host *E. coli* and their resistance plasmids, and therefore is a great improvement on phenotyping methods currently used for reporting AMR trends across Europe by EU agencies. The use of hybrid sequencing contributed further to this characterisation because it provided a greater chance of circularising plasmids more accurately than short-read data alone, identifying the diversity within these mobile-genetic elements, to help monitor their stability and propensity to transfer between different hosts. The reduction of sequencing costs, improved turn-around-time, objectivity and ease of sharing data beyond just AMR profiles has led to the use of WGS for monitoring of key organisms considered by EFSA, with optional sequencing and submission of results in 2021, and mandatory WGS monitoring by 2026. However, it can be important to continue MIC alongside the WGS, at least for a proportion of isolates, because it allows the training of AMR reference databases and inclusion of any gene omissions. Furthermore, discrepancies between the two methods can aid the discovery of new genes or novel AMR genes, and the development of more accurate rules for gene presence.

Although different commercial ESBL agars were used in this study, we believe any variation in selection of isolates between the ESBL agars should be less than between ESBL and McC + CTX agar. This is because McC + CTX allows for the growth of organisms that harbour AmpC, ESBL or carbapenemases, however the ESBL agars are intended to select for only ESBL producers and not AmpC producers. This was demonstrated by the genotypes of the respective isolates from each agar with the main differences between isolates recovered being the increased recovery of isolates from McC + CTX agar with *bla*_CMY-2_ or AmpC mutations instead of an ESBL genotype and a lower proportion of MDR genotypes. In contrast, isolates from ESBL agars mostly harboured an ESBL genotype and had a greater probability of genetic linkage with other AMR genes, and thus were MDR. These results highlight the influence of selective agars on the isolates cultured from the same sample and the diversity of resistance genes they harbour, and we therefore recommend the use of both for surveillance. A further benefit of including both agar types, is that a greater proportion of the bacterial population is likely to be sampled, as suggested by the phylogenetic tree, so important isolates such as the ESBL harbouring ST131 C1-M27 pathogen, which was recovered from the additional ESBL agar not mandated by the EU^25^, are not missed.

Genes belonging to the *bla*_CTX-M_ family were the most common ESBL genes from both agar types (McC + CTX or commercial ESBL agar); *bla*_SHV-12_, was the only other ESBL gene present in a handful of isolates from each year. This concurs with the findings of a Dutch study of *E. coli* from livestock from 2007–2017, which found *bla*_CTX-M_ genes were most common and *bla*_SHV-12_ was present at a lower frequency^26^. Unsurprisingly, *bla*_CTX-M-1_, commonly associated with livestock^27^, was dominant in all years of the study; it’s prevalence from a range of sources has been noted previously^7,26^. IncI1 replicon bearing plasmids were most often associated with *bla*_CTX-M-1_ genes, with 43% of *bla*_CTX-M-1_ isolates harbouring genes showing > 99% identity with pESBL138. The presence of this plasmid in isolates recovered over a five-year period demonstrated its stability, which was unusual compared to the majority of plasmids circularised in this study, that were only found in a handful of isolates, with exception of another *bla*_CTX-M-1_ plasmid, pPE13096. Plasmid stability has been noted in studies of broilers, so this may not be a novel observation^26,28^. However, due to short-read WGS data being used for the majority of isolates in this study, a full plasmid comparison was difficult and it requires further long-read sequencing to determine the variability at minutiae of the plasmids recovered; which was too costly for us to undertake currently. Excluding beta-lactams, isolates from all years showed the highest proportion of genotypic co-resistance to the same three antimicrobials: sulfamethoxazole, tetracycline and trimethoprim, which since 2013 are some of the most sold antimicrobials for animal therapeutics (UK-VARSS 2017, 2018). Resistance to these antimicrobials persisted although results from monitoring showed a decrease in 2017 compared to 2015 in *E. coli* isolated from pigs at slaughter (UK-VARSS 2017, 2018). The co-resistance, resulting in MDR, is likely driven by co-location of *sul*, *tet* and *dfrA* gene variants on plasmids such as IncI1 carrying *bla*_CTX-M-1_ type ESBL genes, as shown in this study. There was a slight decrease in the proportion of MDR isolates in the McC + CTX agar from 2015 to 2017, which coincided with a 50% reduction in antibiotic sales approved for usage in pigs and poultry, and 48% reduction in antibiotics approved for pigs only, from 2013 to 2017^17^. However, there was an increase in the level of MDR from the ESBL isolates from 2013 to 2017. This could be attributed to the MDR rules that are stipulated by EFSA when analysing ESBL and AmpC producing isolates. As ESBL and AmpC producing isolates are also resistant to aminopenicillins (ampicillin), they automatically fulfil two of the three groups required for MDR designation, and as ESBL isolates are more likely to harbour additional resistance genes on plasmids, these isolates are more prone to MDR designation. However, if ampicillin was combined with cefotaxime and ceftazidime to form a single group, a 3–11% reduction in the proportion of MDR would result. Notably, resistance genes to the highest priority-critically important antimicrobials (HP-CIAs) colistin, meropenem and tigecycline not recommended for use in food-producing animals were not detected in any isolates. However, genes conferring resistance to other important antimicrobials such as gentamicin, rifampicin, azithromycin, fosfomycin and ciprofloxacin were detected in multiple isolates. Some of these resistances are not novel, for example, gentamicin and ciprofloxacin resistance have previously been reported in *E. coli* from livestock in Europe^29,30^. Although, identification of *fosA3*, a gene rare in Europe, in five isolates from all years sampled was surprising as this gene is more commonly associated with Asia^31^. *fosA3* was first reported in Europe in 2013 from a migratory bird in Germany, where the gene was located on a MDR IncA/C plasmid containing *bla*_NDM-1_^32^_._ Resistance to antibiotics less commonly used in human medicine but used in treating livestock, such as spectinomycin, was common in all years.

One limitation of this study is that it focussed only on *E. coli* isolates collected from selective media and not on *E. coli* collected from non-selective media from EU surveillance. This only allowed investigation into trends of *E. coli* that are ESBL/AmpC producers and their co-resistances, and not the general resistance trends of all *E. coli* that may be present in livestock and probably harbour other antimicrobial groups. So isolates that may harbour resistances to other HP-CIAs but are not ESCs may be have been missed, as demonstrated during the retrospective *mcr-1* investigations^30,33,34^.

In conclusion, this study demonstrated how the availability of WGS data from both short and long read sequences in bacteria collected from national surveillance activities allowed in-depth scrutiny of resistance trends and dissemination of plasmids, which generally harbour AMR genes. Such analysis if incorporated into surveillance activities would provide a wealth of data to help monitor long-term national AMR trends more accurately, including rapid retrospective analysis of a particular gene, ST or AMR profile. Furthermore, having this genomic information provides enormous value for rapid retrospective molecular characterisation to determine prevalence and transmission, and facilitates comparison with new or current datasets in the event on an outbreak.

## Methods

### DNA extraction of *E. coli* isolated from pig caecal contents

Archived *E. coli* isolated from pig caeca in 2013 using selective agar (CHROMagar CTX or Oxoid Brilliance ESBL) originated from a nationwide pig study by Randall et al*.* 2014^15^ DNA from these were isolated using the QuickGene system (Fujifilm, Japan), and an MP FastPrep (MP Biomedicals, USA), for additional mechanical lysis. The 2015 and 2017 isolates originated from archived *E. coli* previously isolated from the EU mandatory monitoring of AMR in zoonotic and commensal bacteria, outlined by the Commission Implementing Decision 2013/652/EU and the MICs of these isolates to 16 antimicrobials recommended by EFSA were obtained by following EU monitoring protocols^2^. They were grown on either MacConkey supplemented with 1 mg/L cefotaxime (McC + CTX) or CHROMagar ESBL, depending on which the *E. coli* had originally been isolated on. DNA was extracted from the 2015 isolates using a crude boilate as described previously^30,33^. For the 2017 isolates, a single colony was used to inoculate a 3 mL overnight culture in LB. The overnight culture was the starting material for the MagMax core nucleic acid purification kit and extracted with a Kingfisher Flex system (Thermofisher, USA) using the heated script protocol. For minION Nanopore sequencing, DNA was extracted using a Qiagen genomic 100/G tip, barcoded using a rapid barcoding kit, used as per manufacturer’s instructions.

### WGS and analysis

The 2013 (n = 185) isolates were sequenced with an Illumina HiSeq, whilst the 2015 (n = 188) and 2017 (n = 128) isolates were sequenced using an Ilumina MiSeq or NextSeq. A subset of six isolates were sequenced on an Oxford Nanopore minION. SPAdes 3.11.1 was used for assembling the short-read data; Unicycler for the short and long-read hybrid data; and Prokka 1.11 for annotation^35–37^. MLST of the isolates was completed with SRST2, and presence of AMR genes and plasmids determined with APHA SeqFinder and Abricate^30,38,39^. The isolates were aligned to *E. coli* K12 with Snippy, Gubbins was used to remove recombination regions, and RAxML was used to build a phylogeny (trees were annotated with iTOL), and Kraken was used to for taxonomic classification^40–43^. Circularised plasmid sequences were compared to the genome assemblies of all other isolates using fastANI^44^ and blastN for comparing to those held by NCBI. pMLST was used to determine the plasmid ST^45^. An isolate was considered MDR if it contained genes conferring resistance to three or more different groups of antimicrobials listed in the scientific report published by EFSA in 2018 ^46^. Following the EFSA MDR stipulations, cefotaxime and ceftazidime were treated as a single group, as were ciprofloxacin and nalidixic acid. Genotypic resistance was compared to previously generated phenotypic MIC data for the 2015^18^ and 2017^17^ McC + CTX isolates using methods outlined in Stubberfield et al.^16^.

## Supplementary information

Supplementary Information 1.

Supplementary Information 2.

## Data Availability

All raw Illumina and Nanopore data produced in this study have been deposited under PRJEB34493. Plasmid sequences have been deposited under accession numbers MW077910-MW077917.
