# Supplementary material for: The importance of using whole genome sequencing and extended spectrum beta-lactamase selective media when monitoring antimicrobial resistance
Source: Sci Rep. 2020 Nov 16;10:19880. doi: 10.1038/s41598-020-76877-7 (PMC7670430; doi:10.1038/s41598-020-76877-7)
Supplement: Supplementary file 2 — Supplementary Information 2. [file 41598_2020_76877_MOESM2_ESM.pdf]

The importance of using whole genome sequencing and extended spectrum beta-lactamase selective media when monitoring antimicrobial resistance.

Nicholas Duggett, Manal AbuOun, Luke Randall, Robert Horton, Fabrizio Lemma, Jon Rogers, Derrick Crook, Chris Teale, Muna F. Anjum.

Supplementary material

**Antimicrobial**	**Gene**
Azithromycin	*ermB, mefB, mphA, mphB*
Cefotaxime, Ceftazidime, Ampicillin	*LAP-2, OXA-1, OXA-10, TEM-1a, TEM-1b, TEM-1c, TEM-1d, CMY-2, CTX-M-1, CTX-M-11, CTX-M-14, CTX-M-15, CTX-M-16, CTX-M-2, CTX-M-27, CTX-M-3, CTX-M-32, CTX-M-55, CTX-M-65, DHA-1, MOX-9, SHV-12, ampC* promoter mutation
Chloramphenicol	*catA1, catA2, catB7, cml, floR*
Ciprofloxacin, Nalidixic Acid	*gyrA* mutation, *parC* mutation, *qnrB1, qnrB19, qnrB4, qnrS1, aac6-lb-cr*
Gentamicin	*aac3-pEK516, aac3-IVa, aac3-VIa_c, aac6-Ib3, aph3-IIb, aac3-IId*
Sulphamethoxazole	*sul1, sul2, sul3*
Tetracycline	*tet-AB, tet-A, tet-B, tet-C*
Trimethoprim	*dfrA1, dfrA12, dfrA13, dfrA14, dfrA17, dfrA21, dfrA5, dfrA7, dfrA8, dfrB1*
Fosfomycin	*fosA3*
Kanamycin	*aph3-Ia, aph3-Ic*
Lincomycin	*lnuF*
Rifampicin	*arr-2*
Streptomycin and Spectinomycin	*strA, strB, aadA12, aadA17, aadA1b, aadA2, aadA22_b, aadA23, aadA24, aadA3, aadA5, aadA6/aadA10, aadA8, ant3-1a*
Streptothricin	*sat2A*

Table S1 – List of antimicrobials and their constituent genes that were identified in this study

	Gene	2013	2015		2017		Associated Plasmid Inc types identified
		CA-CTX and ESBL Brilliance (n=185/637)	ESBL Brilliance (n=96/313)	McC+CTX (n=89/313)	CA-ESBL (n=53/347)	McC+CTX (n=75/347)	
**ESBL**	CTX-M-1	76	64	51	64	44	IncI1, IncFII, IncB/O/K/Z
	CTX-M-2	1	4	2	0	1	-
	CTX-M-3	1	2	0	0	0	IncB/O/K/Z
	CTX-M-14	2	5	3	4	7	IncB/O/K/Z
	CTX-M-15	9	14	9	8	11	IncI1, IncFI, IncY
	CTX-M-27	1	0	1	2	0	-
	CTX-M-32	1	1	1	4	2	IncFI
	CTX-M-55	2	4	7	2	5	-
	CTX-M-65	0	0	1	2	0	IncFI
	CTX-M-115	0	0	0	2	1	-
	SHV-12	8	6	4	9	3	IncI1, IncX3
**AmpC**	CMY-2	0	0	9	1	16	IncI1
	DHA-1	0	0	3	0	0	-
	MOX-9	0	0	0	1	0	-
	AmpC mutation	1	1	8	2	11	N/A

Table S2 – AmpC/ESBL genes per year with the number of isolates from the total number of caecal samples recovered for each year.

Antimicrobial		2013	2015		2017	
		**CA-CTX and ESBL Brilliance (n=185/637)**	**ESBL Brilliance (n=96/313)**	**McC+CTX (n=89/313)**	**CA-ESBL (n=53/347)**	**McC+CTX (n=75/347)**
EFSA	Ampicillin	100	100	100	100	100
	Cefotaxime/Ceftazidime	100	100	100	100	100
	Azithromycin	19	9	15	11	9
	Chloramphenicol	23	26	28	21	21
	Ciprofloxacin/Nalidixic acid	19	27	26	25	17
	Colistin	0	0	0	0	0
	Gentamicin	16	19	19	8	12
	Meropenem	0	0	0	0	0
	Sulphamethoxazole	85	88	79	83	75
	Tetracycline	75	79	79	83	67
	Tigecycline	0	0	0	0	0
	Trimethoprim	69	69	70	74	75
**MDR^**		98	95	94	100	89
Non-EFSA	Fosfomycin	2	1	1	0	3
	Kanamycin	5	5	7	4	7
	Lincomycin	2	2	4	2	1
	Spectinomycin/Streptomycin	81	80	76	85	80
	Streptothricin	7	5	4	13	9

Table S3 – Number of isolates out of total number of caecal samples examined per year/agar type with the proportion of isolates that contained genes that can confer resistance to the ESFA antimicrobial monitoring panel, other antimicrobials and the proportion of isolates that were MDR. ^MDR was defined as the presence of genes conferring resistance to three or more of the EFSA panel groups as listed in the table.

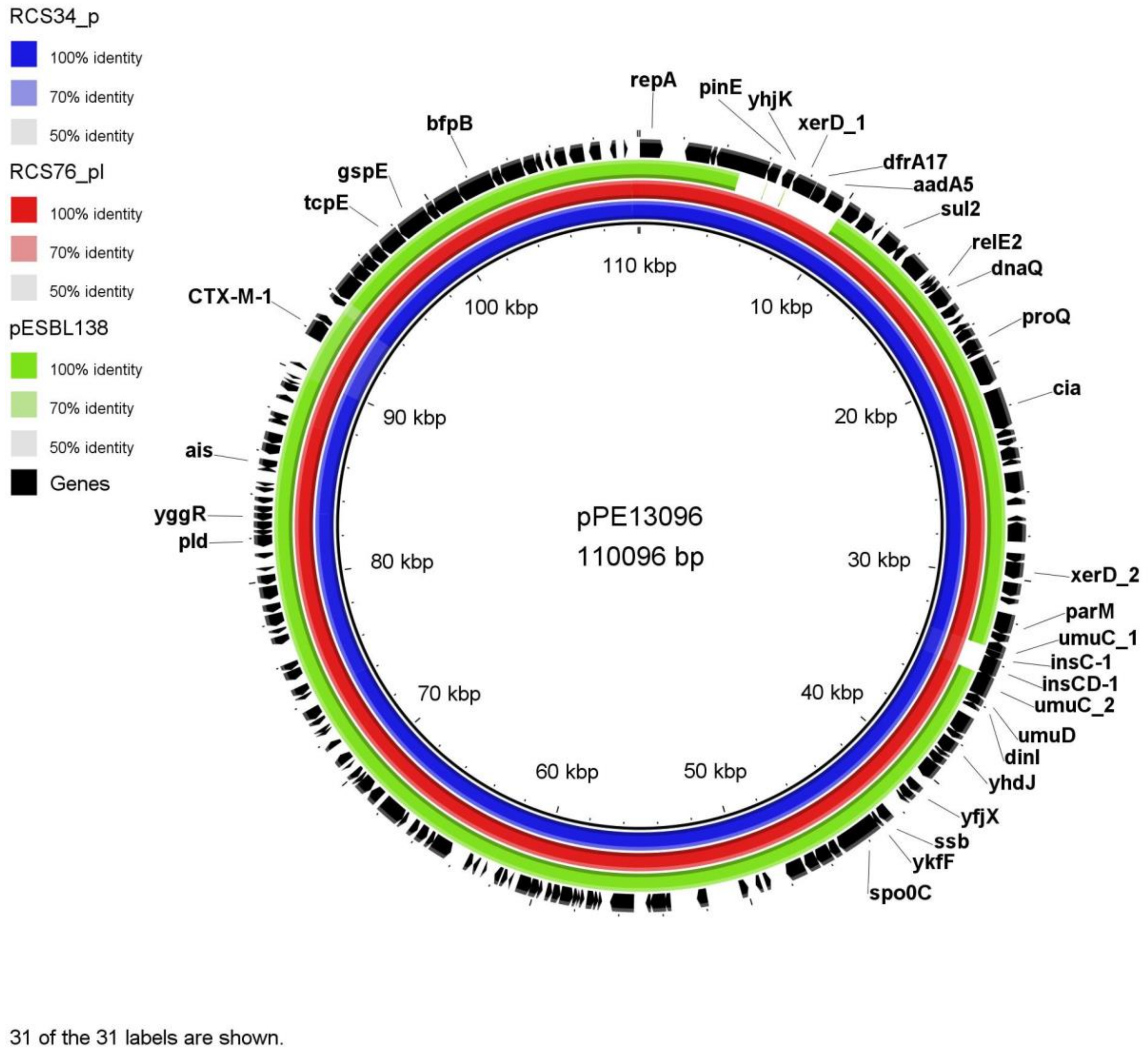

Figure S1 – Comparison of the bigger IncI1 plasmid (pPE13096) with those found in NCBI (RCS34_p and RCS76_pl) and the smaller, but more common, IncI1 plasmids in this study (pESBL138)

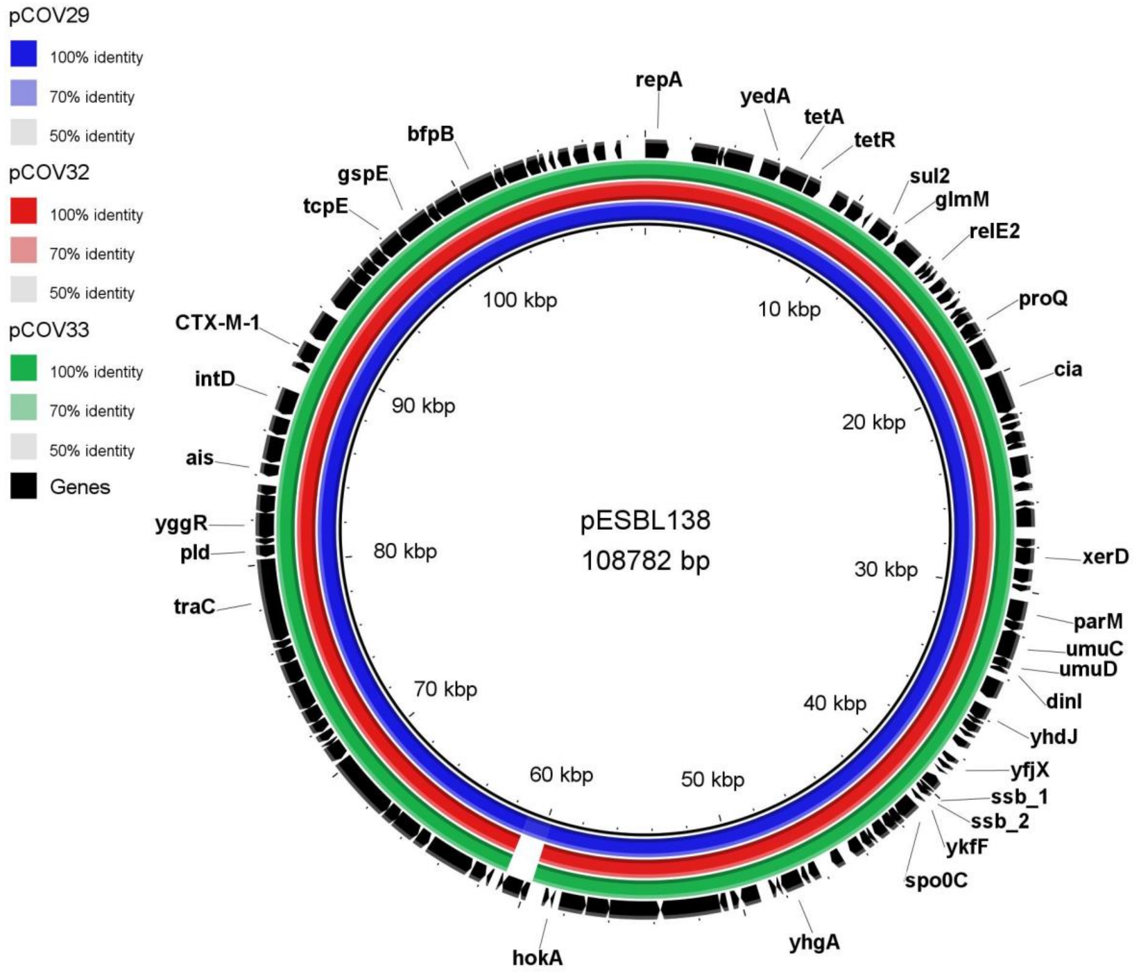

31 of the 31 labels are shown.

Figure S2 – pESBL138 compared to two plasmids isolated from broilers in France

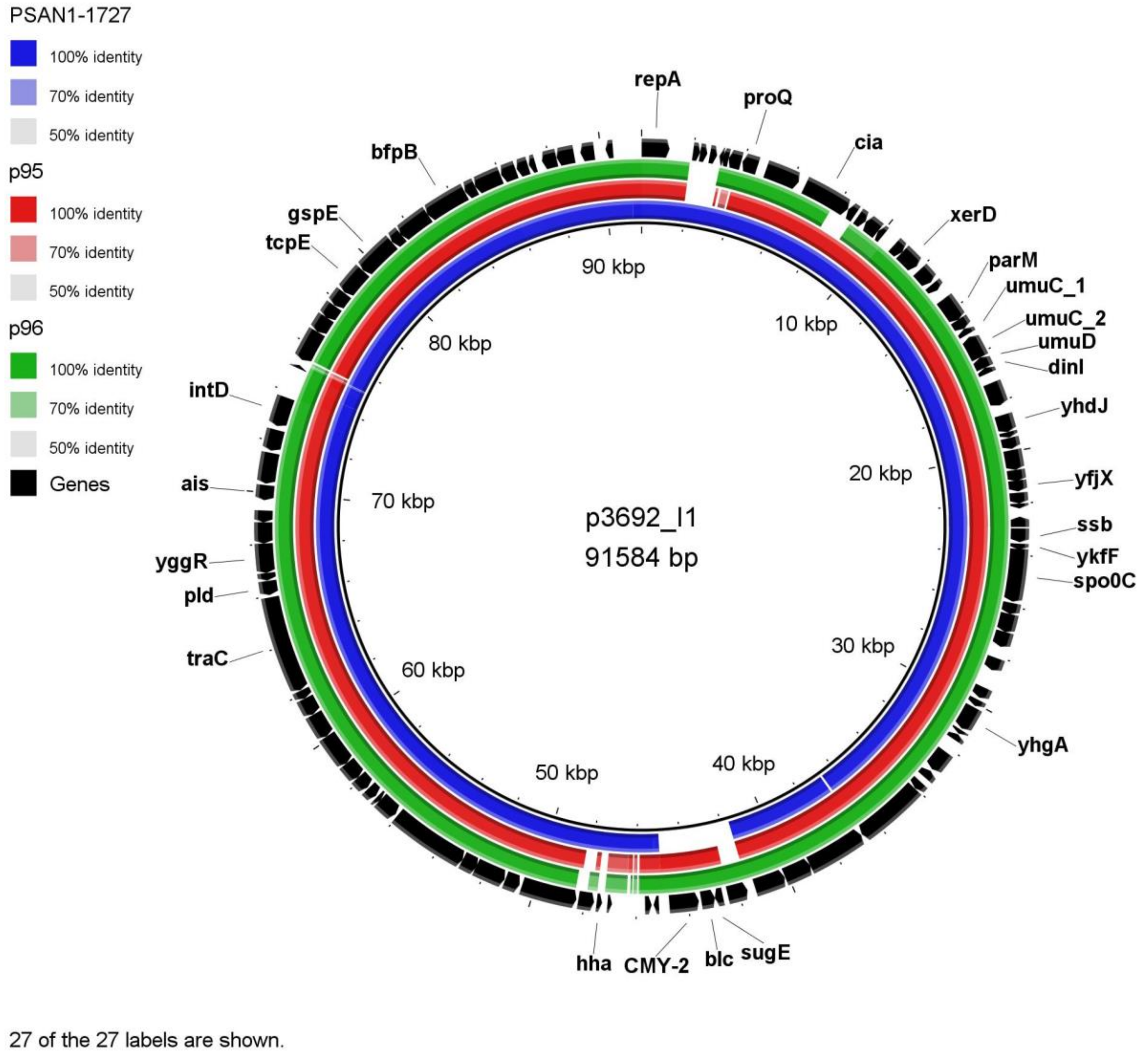

Figure S3 – p3682 compared to three plasmids. Of the plasmids (blue ring) was from a Salmonella from the USA, the backbone was the same but it was missing CMY-2.

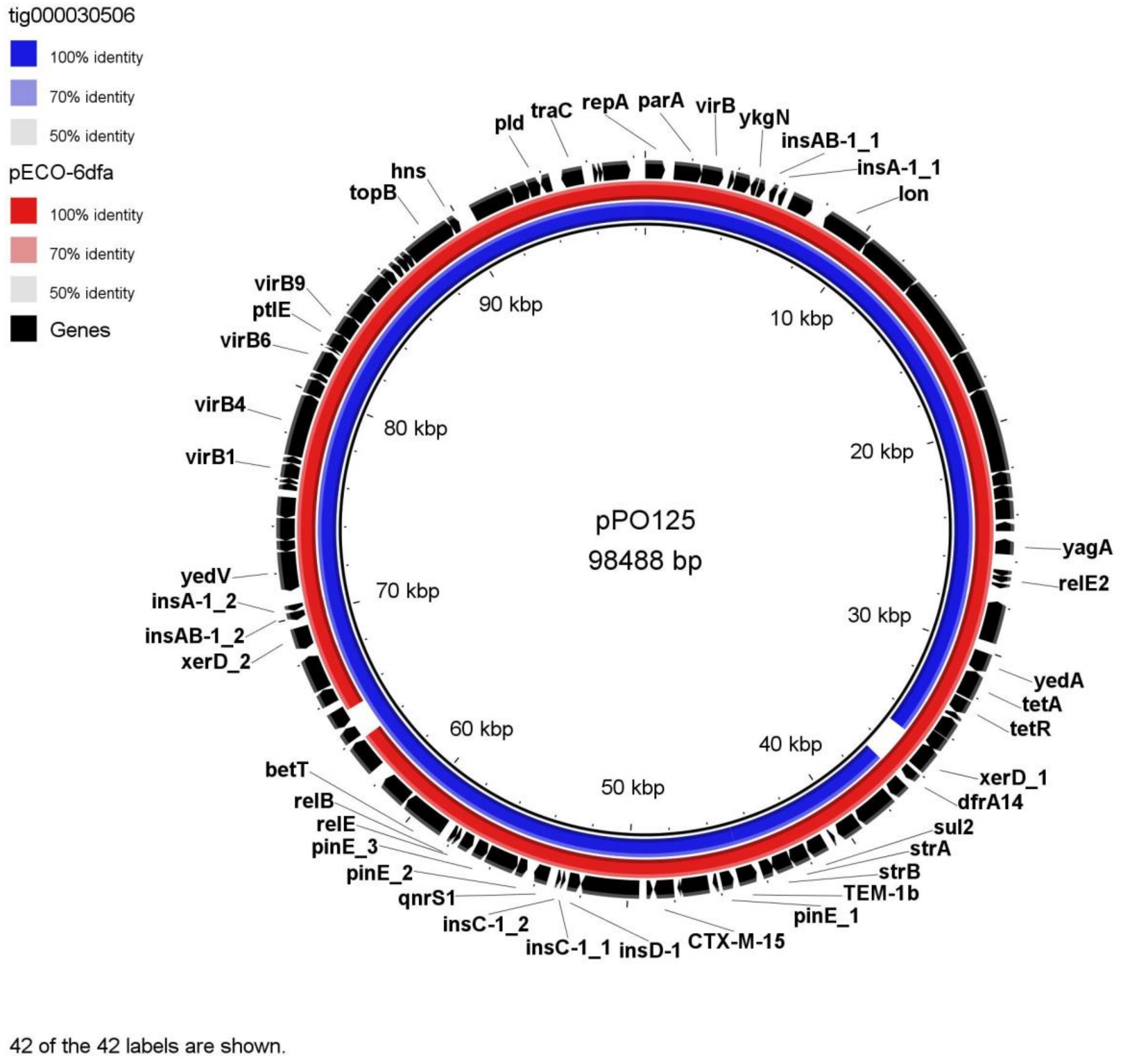

Figure S4 – bla-CTX-M-15 on an IncY plasmid showing high similarity to isolates from an E. coli recovered from hospital plumbing from the USA.

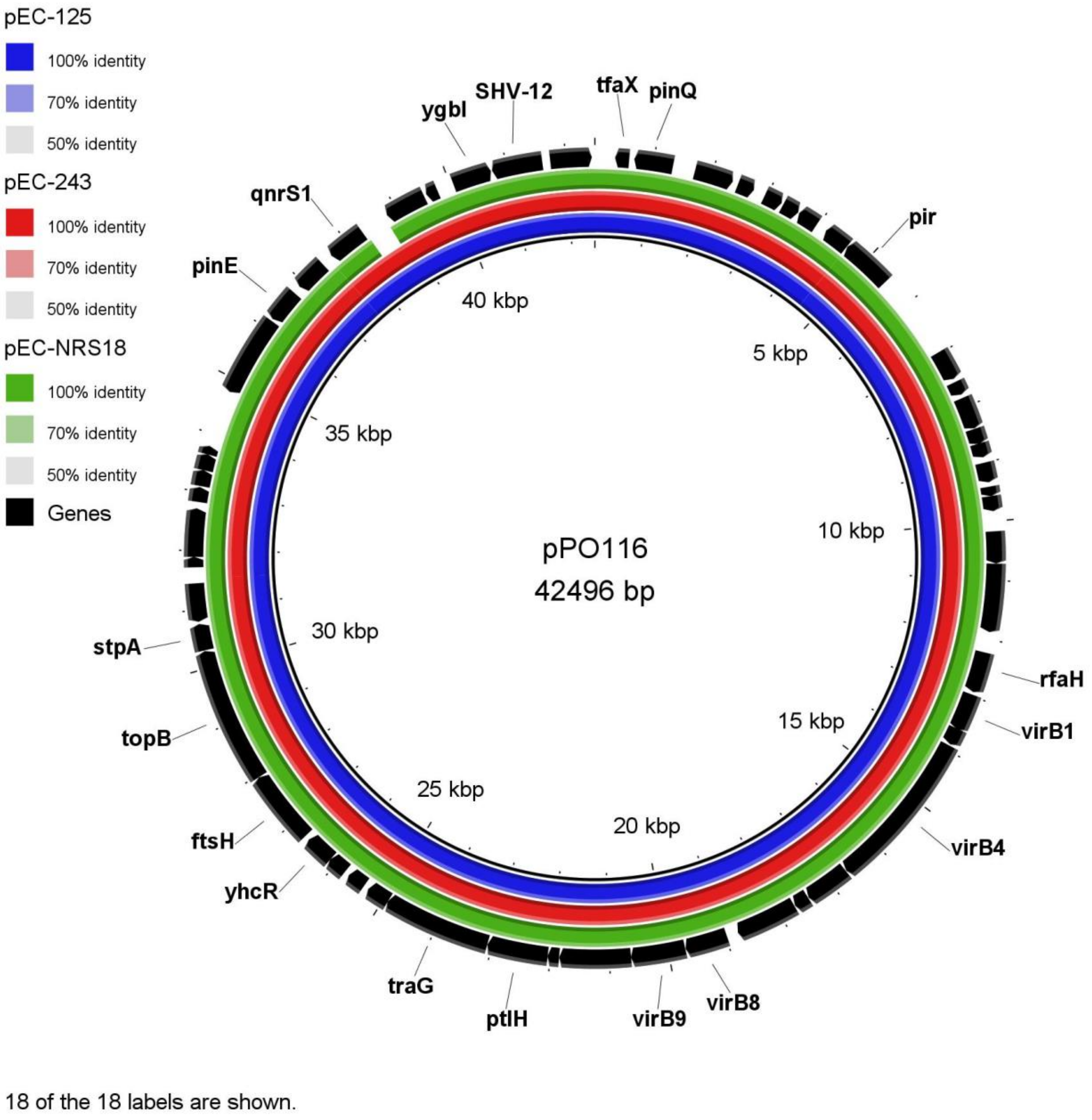

Figure S5 – An IncX3 plasmid showing similarity to ones from the Netherlands that were isolated from a human and two from chicken faeces.
